# A potential regulatory loop between Lin28B:miR-212 in androgen-independent prostate cancer

**DOI:** 10.3892/ijo.2014.2647

**Published:** 2014-09-09

**Authors:** EMMA BORREGO-DIAZ, BENJAMIN C. POWERS, VUGAR AZIZOV, SCOTT LOVELL, RUBEN REYES, BRADLEY CHAPMAN, OSSAMA TAWFIK, DOUGLAS McGREGOR, FRANCISCO J. DIAZ, XINKUN WANG, PETER VAN VELDHUIZEN

**Affiliations:** 1 Division of Hematology/Oncology, Department of Internal Medicine, University of Kansas Medical Center, Westwood, KS 66205, USA; 2 University of Kansas Cancer Center, Kansas City, KS 66160, USA; 3 Veterans Administration Medical Center, Kansas City, MO 64128, USA; 4 Protein Structure Laboratory, Del Shankel Structural Biology Center, University of Kansas, Main Campus, Lawrence, KS 66047, USA; 5 Department of Pathology and Laboratory Medicine, University of Kansas Medical Center, Kansas City, KS 66160, USA; 6 Department of Biostatistics, University of Kansas Medical Center, Kansas City, KS 66160, USA; 7 Genomic Facility, University of Kansas, Main Campus, Lawrence, KS 66047, USA

**Keywords:** Lin28B-miR-212-c-Myc pathway, miR-212, Lin28B silencing, androgen-independent prostate cancer, microRNA regulation, c-Myc downregulation

## Abstract

Lin28 is a family of RNA binding proteins and microRNA regulators. Two members of this family have been identified: Lin28A and Lin28B, which are encoded by genes localized in different chromosomes but share a high degree of sequence identity. The role of Lin28B in androgen-independent prostate cancer (AIPC) is not well understood. Lin28B is expressed in all grades of prostatic carcinomas and prostate cancer cell lines, but not in normal prostate tissue. In this study we found that Lin28B co-localized in the nucleus and cytoplasm of the DU145 AIPC. The expression of Lin28B protein positively correlated with the expression of the c-Myc protein in the prostate cancer cell lines and silencing of Lin28B also correlated with a lower expression of the c-Myc protein, but not with the downregulation of c-Myc messenger RNA (mRNA) in the DU145 AIPC cells. We hypothesized that Lin28B regulates the expression of c-Myc protein by altering intermediate c-Myc suppressors. Therefore, a microRNA profile of DU145 cells was performed after Lin28B siRNA silencing. Nineteen microRNAs were upregulated and eleven microRNAs were downregulated. The most upregulated microRNAs were miR-212 and miR-2278. Prior reports have found that miR-212 is suppressed in prostate cancer. We then ran TargetScan software to find potential target mRNAs of miR-212 and miR-2278, and it predicted Lin28B mRNA as a potential target of miR-212, but not miR-2278. TargetScan also predicted that c-Myc mRNA is not a potential target of miR-212 or miR-2278. These observations suggest that Lin28B:miR-212 may work as a regulatory loop in androgen-independent prostate cancer. Furthermore, we report a predictive 2-fold symmetric model generated by the superposition of the Lin28A structure onto the I-TASSER model of Lin28B. This structural model of Lin28B suggests that it shows unique microRNA binding characteristics. Thus, if Lin28B were to bind miRNAs in a manner similar to Lin28A, conformational changes would be necessary to prevent steric clashes in the C-terminal and linker regions between the CSD and ZNF domains.

## Introduction

Prostate cancer is the most common cancer in men in Western countries (1). Castrate-resistant or androgen-independent prostate cancer (AIPC) is a more aggressive form seen later in the disease process, and by definition, is more resistant to therapeutic intervention (2). Many of the general treatment strategies for this type of prostate cancer involve androgen deprivation by a variety of strategies such as luteinizing hormone-releasing hormone agonists, anti-androgens, estrogens, orchiectomy and drugs preventing both intratumoral and adrenal gland androgen production (3). Since almost all prostate cancers eventually develop castrate resistance it is critically important to understand the mechanisms leading to the progression to AIPC, with the hope of discovering new effective therapeutic methods. In that direction, microRNAs and their regulators have become an attractive area of research.

MicroRNAs are small non-coding molecules of RNA (4). They have been shown to regulate gene expression of proteins that participate in tumorigenesis, cell cycle regulation, stress response, inflammation, differentiation, apoptosis and metastasis (4). MicroRNAs are conserved from plants to human and are encoded by their own genes. miRNA genes are localized in separate gene loci, or they can be found within introns and exons of other genes. The maturation process of microRNAs implicates transcription, nuclear export and cleavage leading to 18–22 nucleotide double-stranded RNA molecules that enter a cytoplasmic protein complex to regulate gene expression at the post-transcriptional level (5,6). miRNAs can modulate entire gene programs. They do not intercept a single target as in the case of selective protein inhibitors (4). Examinations of the regulatory mechanism of the genome to discover RNAs that can interfere between transcription and translation stages of protein synthesis are necessary to understand the progression of androgen-independent prostate cancer and equally important to develop new therapeutic procedures to treat this disease.

The Lin28 protein family acts as RNA binding proteins and microRNA regulators (7,8). The genes that code for human Lin28A and Lin28B, the two known members of this protein family, are localized on different chromosomes, 1p36.1 (Gene ID 79727) and 6q21 (Gene ID 389421), respectively. Following their discovery, published literature clearly shows that Lin28A and Lin28B have different cellular functions (9). Lin28B has been shown to be tumorigenic in a prostate cancer mouse model (10) but the role of Lin28B in androgen-independent prostate cancer is unknown.

Lin28B is expressed in all grades of prostatic carcinomas and prostate cancer cell lines, but not in normal prostate tissue. We found that Lin28B co-localized in the nucleus and cytoplasm of the DU145 androgen-independent prostate cancer cells. Also, the expression of Lin28B protein positively correlated with the expression of the c-Myc protein in prostate cancer cells. Furthermore, the silencing of Lin28B also correlated with a lower expression of c-Myc protein, but not with the downregulation of c-Myc messenger RNA. MiR-212 and miR-2278 seems to be the most upregulated microRNAs upon Lin28B silencing by siRNA. Prior reports have found that miR-212 is suppressed in prostate cancer tissues but not in normal prostate tissues (9). Therefore, our results may suggest that Lin28B is an oncogene suppressing miR-212 expression in androgen-independent prostate cancer cells. On the other hand, miR-2278 has not been study in cancer or other disease states. Our analysis using the Target Scan shows that only miR-212 could target the mRNA of Lin28B. Members of the Lin28 protein family are RNA binding proteins that act as regulators of microRNAs (7) and crystallography and modeling work in our laboratory suggests that Lin28B has unique and specific interactions with microRNAs.

## Materials and methods

### Cells, antibodies and the chemical inhibitor

Human prostate cancer cell lines (VCaP, vertebral metastasis from prostate; LNCaP, prostate left supraclavicular lymph node; PC3, prostate-bone; and DU145, prostate-brain) were purchased from American Type Culture Collection (ATCC, Manassas, VA, USA; catalog nos. CRL-2876, CRL-1740, CRL-1435 and HTB-81, respectively). VCaP, PC3 and DU145 cells were maintained in Dulbecco’s modified Eagle’s medium (DMEM). RPMI-1640 medium was used for LNCaP cells. In all cases, the medium was supplemented with 10% fetal bovine serum (FBS) and 1% penicillin/streptomycin solution.

Primary antibodies used for fluorescent-activated cell sorting (FACS) and western blotting were human Lin28B and β-actin (Cell Signaling, Danvers, MA, USA; catalog nos. 4196S, 2496S and 3700, respectively). The human c-Myc antibody was purchased from Santa Cruz Biotechnology (Dallas, TX, USA; catalog no. sc-788). Lin28B antibody used for the immunofluorescence study was purchased from Santa Cruz Biotechnology (catalog no. sc-130802).

### Transfection of prostate cancer cells using siRNA

For the transient transfection experiments, the DU145 AIPC cells were transfected with ON-TARGETplus siRNA which is an exceptional choice for optimal gene silencing and it may reduce off-targets up to 90% compared to unmodified siRNA. We transfected DU145 AIPC cells with the siRNA ON-TARGETplus smart pool, human Lin28B 3ÚTR/ORF [a pool of four different siRNAs (GGAUAUUCCAGUCGAUGUA, GCCCAUAAGUGUUAAUAGA, CAAGCGUAUUGCAGCAUUA and CCAGAGAGCUAGAAGUAUU)] or siRNA ON-TARGETplus non-targeting pool (control for the transfection/silencing experiment), from Thermo Scientific Rockford, IL, USA; catalog nos. L-028584-01-0005 or D-001810-10-05, respectively. The Block-it transfection kit (Invitrogen, Carlsbad, CA, USA; catalog no. 13570-070) was used according to manufacturer suggestions.

### FACS analysis and western blot examination to evaluate Lin28B expression in prostate cancer cell lines

FACS and western blot examinations were performed on LNCaP, VCaP, PC3 and DU145 prostate cancer cell lines to determine the expression of Lin28B and c-Myc proteins. In addition, FACS and western blot examinations were made on DU145 cells transfected.

### FACS

The transfected DU145 AIPC cells were treated with trypsin (Sigma-Aldrich, St. Louis, MO, USA) and washed using cold 1× PBS. 500,000 cells were fixed in 2% methanol-free formaldehyde (Thermo Scientific) and permeabilized with BD IntraSure kit (Pharmingen, Pasadena, CA, USA; catalog no. 641776). Then, cells were exposed to primary antibodies (Lin28B and c-Myc) for 1 h at room temperature, followed by three washes with cold 1× PBS. Next, cells were stained with Alexa Fluor-488 goat anti-rabbit IgG (H+L) antibody (Molecular Probe, Carlsbad, CA, USA; catalog no. A11008). Finally, cells were washed three times with cold 1× PBS and suspended in 250 μl of 1× PBS. The samples were analyzed by FACS using the LSR II instrument (BD Biosciences, San Jose, CA, USA) at the core facility of the University of Kansas Medical Center. All data were analyzed by FlowJo software.

### Western blotting

Cell lysates from the transfected DU145 AIPC cells were prepared by the addition of lysis buffer, containing 50 mm Tris-HCl, pH 8.0, 0.1% SDS, 150 mM NaCl, 1% nonidet P-40, and a protease inhibitor combination including 1 μg/ml aprotinin, 1 μg of leupeptin and 1.0 mM PMSF. Equal amounts of protein were loaded to 10% SDS-PAGE and transferred to a nitrocellulose membrane. Membranes were incubated with specific primary antibodies overnight followed by secondary antibodies. Super Signal ULTRA Chemiluminescent Substrate (Thermo Scientific) quantitated the signals by using ID Image Analysis Software Version 3.6 (Eastman Kodak Co., Rochester, NY, USA).

### RNA isolation

Total RNA was extracted from the DU145 AIPC cells upon transfection with and without Lin28B-siRNA using TRIzol (Invitrogen; catalog no. 15596018) following the manufacturer’s protocol.

### GeneChip-miRNA 2.0 array

MicroRNA profiling was performed at the Genomics Facility at the University of Kansas (Lawrence, KS) using GeneChip-miRNA 2.0 array (Affymetrix, Santa Clara, CA, USA; catalog no. 901754). For miRNA sample labeling, the Genisphere FlashTag Biotin HSR RNA Labeling kit, GeneChip Eukaryotic Hybridization Control kit and GeneChip Hybridization, Wash and Stain kit were used (Affymetrix; catalog nos. 901910, 900454 and 900720, respectively).

### Real-time PCR

For validation of the overexpression of miR-212, a Taqman microRNA reverse transcription kit was used to prepare cDNA from the total RNA. The cDNA was used to run the real-time PCR using Taqman universal PCR and a Taqman microRNA assay kit (Applied Biosystem Step One real-time PCR system). PCR was completed for 15 sec at 95°C and 1 min at 60°C for 40 cycles. CT values for miR-212 were normalized to control RNU58A by subtracting the average CT value for each sample. The relative quantification values for miR-212 in each sample were determined using the 2^−Δ ΔCT^ method (11). Each PCR reaction was performed in at least triplicate (mean ± SD). The fold expression levels for Lin28B and c-Myc once Lin28B was silencing in DU145 prostate cancer cells using siRNA was calculated by ΔΔC_T_ methods using GAPDH as an endogenous control (following the instruction from Applied Biosystems) (11). Relative quantitation of gene expression was analyzed with the comparative C_T_ method (11). Each ΔΔC_T_ was computed as a contrast in a one-way ANOVA analysis, and the p-value testing the significance of the contrast was used to test the significance of the corresponding fold change expression.

The genes targeted by miR-212 were predicted by software packages available online from TargetScan (www.targetscan.org/) and microRNA (www.microrna.org/microrna/home.do).

### Immunofluorescence

Protein expressions were evaluated by immunofluorescence using Abcan protocols. Briefly, paraffin-embedded sections from the prostate tissue array T191 and PR243a (US Biomax, Inc., Rockville, MD, USA) were de-paraffinized in xylene, hydrated with 100% ethanol and 95% ethanol for 5 and 1 min, respectively. Slides were then rinsed in distilled water and samples were washed twice with ice cold PBS. For blocking unspecific binding of the antibodies, samples were incubated with 1% BSA in PBST for 1 h. The anti-Lin28B primary antibody was diluted in the blocking solution and incubated with the samples overnight at 4°C. Slides were washed with 1× PBS and the secondary antibody was added in blocking solution for 1 h at room temperature, followed by three washes with 1× PBS and then the addition of the mounting anti-fade solution with DAPI. The slides were stored in the dark at 4°C. The expression of Lin28B was evaluated in the DU145 prostate cancer cell line using the same protocol for labeling. Fixation and permeabilization of the samples were done using acetone for 10 min at room temperature.

### Structure prediction calculations

Structure prediction calculations were performed using the I-TASSER (12) server without additional constraints or templates. The following amino acid sequence of human Lin28B (NCBI Reference: NP_001004317.1) was submitted online in September 2013: MAEGGASKGGGEEPGKLPEPAEEESQVLRGTGHCKWFNVRMGFGFISMINREGSPLDIPVDVFVHQSKLFMEGFRSLKEGEPVEFTFKKSSKGLESIRVTGPGGSPCLGSERRPKGKTLQKRKPKGDRCYNCGGLDHHAKECSLPPQPKKCHYCQSIMHMVANCPHKNVAQPPASSQGRQEAESQPCTSTLPREVGGGHGCTSPPFPQEARAEISERSGRSPQEASSTKSSIAPEEQSKKGPSVQKRKKT.

## Results

### Lin28B is expressed in prostate cancer tissues

**Figure 1:**
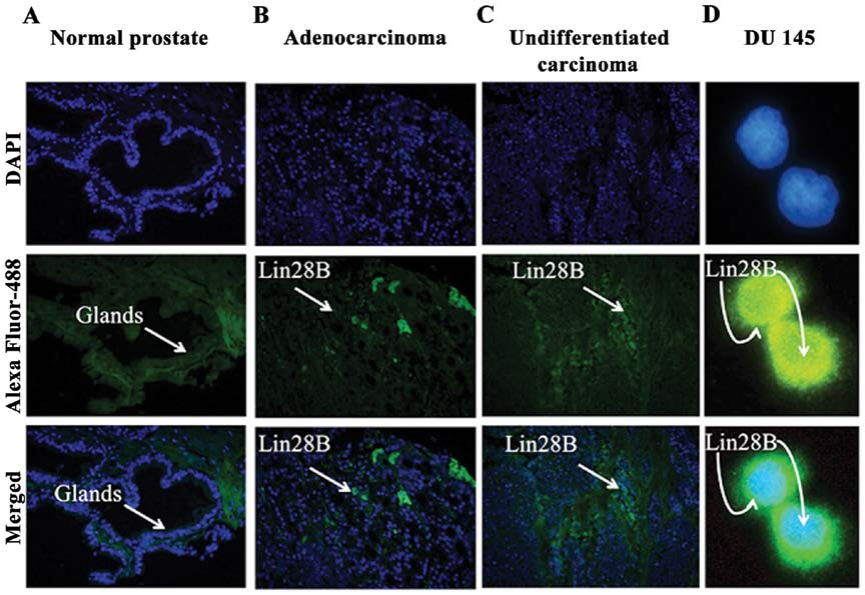
Lin28B expression in human prostate cancer tissues and cellular localization of Lin28B in AIPC cells. (A) Normal prostate tissue. (B) Adenocarcinoma tissue. (C) Undifferentiated carcinoma. (D) Cellular localization of Lin28B in DU145 AIPC cells.

It co-localized in the nucleus and cytoplasm of the DU145 AIPC cells. Two prostate tissue arrays T191 and PR243a from US Biomax, Inc., which includes normal prostate control tissues, well to poorly differentiated adenocarcinomas and undifferentiated carcinoma were used to identify the expression of Lin28B. Lin28B expression was not detected in the glands of normal prostate tissue. Lin28B is expressed in grade 2–3 prostate adenocarcinoma, and undifferentiated grade 4 prostate carcinomas (Fig. 1A–C). Only selected panels from the tissue arrays are shown. Lin28B co-localizes in the nucleus and in the cytoplasm of the DU145 prostate cancer cell line, indicating possible purpose in both cellular compartments (Fig. 1D).

### Lin28B protein expression correlates with c-Myc protein expression in prostate cancer cell lines

**Figure 2:**
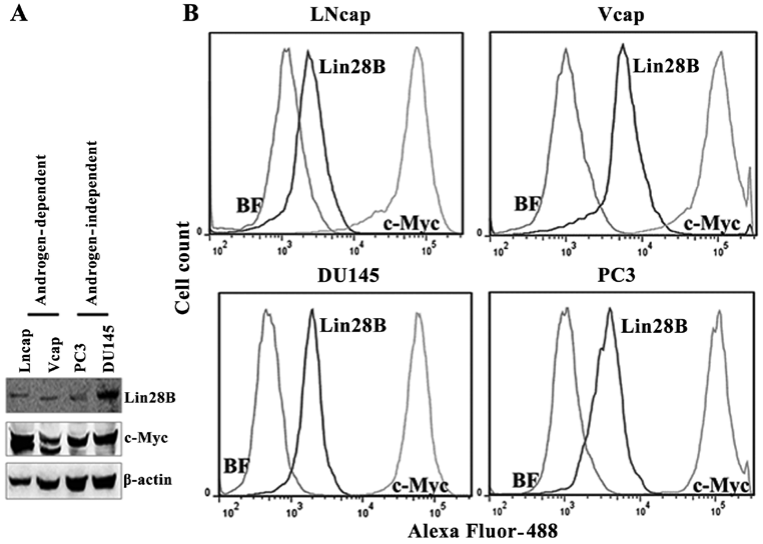
Lin28B and c-Myc expression in the prostate cancer cell lines LnCaP and VCaP (androgen-dependent), PC3 and DU145 (androgen-independent). (A) Determined by western blotting. (B) Determined by flow cytometer (LnCaP and VCaP, DU145 and PC3 prostate cancer cell lines).

Lin28B and c-Myc protein levels were detected by western blotting in androgen-dependent (LNCaP and VCaP) and androgen-independent prostate cancer cell lines (DU145 and PC3) by western blotting, using β-actin to normalize the expression of the proteins. The expression of Lin28B correlates with the expression of c-Myc protein levels in these cell lines. But, the c-Myc antibody detected two bands for c-Myc protein in the two androgen-dependent cell lines and a single band in the androgen-independent cell lines. The quantification of the protein levels for Lin28B and c-Myc was also performed by flow cytometry because western blotting is not a protein quantification tool. The flow cytometry data indicated that Lin28B protein levels are 12.8, 84, 80.9 and 63.7% in LnCaP, VCaP, PC3 and DU145 cells, respectively, in 10,000 cells counted (Fig. 2). The mean fluorescence of cells expressing Lin28B was low (in the range of 10^3^–10^4^) whereas cells expressing c-Myc were bright, in the range of 10^5^ for all the cell lines. This may indicate that copies of Lin28B/cell are lower than c-Myc. The protein levels of c-Myc were the highest in all the cell lines analyzed. Lin28B and c-Myc were detected in all the prostate cancer cells investigated, regardless of androgen-dependence status.

### The silencing of Lin28B in the DU145 AIPC cells correlates with the downregulation of c-Myc protein but not with the downregulation of c-Myc messenger RNA

**Figure 3:**
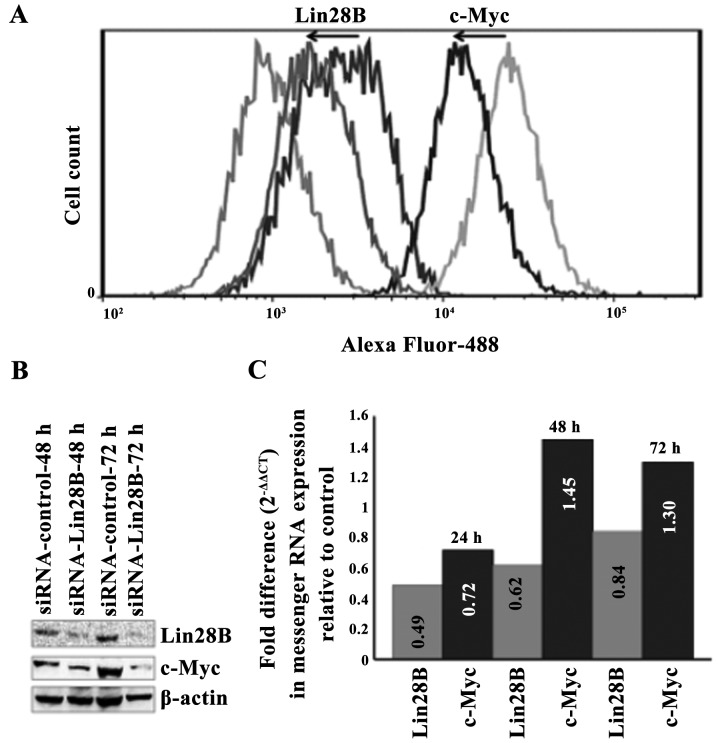
Expression of Lin28B and c-Myc in DU145 AIPC cells upon silencing Lin28B using siRNA. (A) The silencing of Lin28B protein correlates with the downregulation of c-Myc protein at 48 and 72 h as detected by western blotting. (B) Quantification using flow cytometry and data analysis by FlowJo software at 42 h upon transfection with Lin28B siRNA showing background fluorescence (2.4%), Lin28B expression upon Lin28B silencing (32.7%), control expression of Lin28B in DU145 cells (76.8%), c-Myc expression upon Lin28B silencing (58.6%) and control expression of c-Myc in DU145 cells (99.9%). (C) The fold difference in the Lin28B and c-Myc messenger RNA expression relative to the control (2^−ΔΔCT^) upon Lin28B silencing with c-MYC messenger RNA expression slightly decreasing at 24 h (0.72) but recovering by 48 h (1.45).

Lin28B was transiently silenced in the DU145 AIPC cell line using a targeting pool of four siRNAs. The silencing of Lin28B protein correlates with the downregulation of c-Myc protein at 48 and 72 h as detected by western blotting (Fig. 3A). Quantification using flow cytometry and data analysis by FlowJo software also show that the silencing of the Lin28B protein correlates with the downregulation of c-Myc protein at 48 h. The transient silencing of the Lin28B gene decreased Lin28B protein by 42.6% and this compares with the decrease of c-Myc protein by 58.6% at 42 h upon transfection with Lin28B siRNA (Fig. 3B). The level of c-Myc messenger RNA decreased at 24 h after Lin28B gene silencing but it did not reach statistical significance and these levels after 48 h (Fig. 3C), while c-Myc protein remained downregulated at 48 and 72 h.

### Antagonist effect of the RNA binding-microRNA regulator Lin28B protein on miR-212 and miR-2278 microRNAs in DU145 AIPC cells

**Figure 4:**
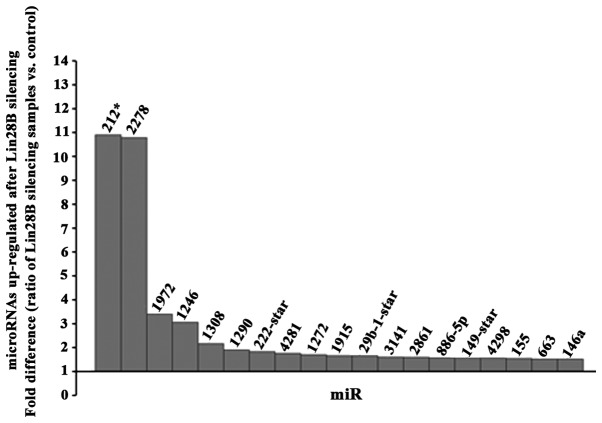
Fold difference (ratio of Lin28B silencing samples vs. control) of microRNAs upregulated in DU145 AIPC cells. MicroRNA profiling was determined by GeneChip-miRNA 2.0 array. A threshold of 1.5-fold change was used.

**Figure 5:**
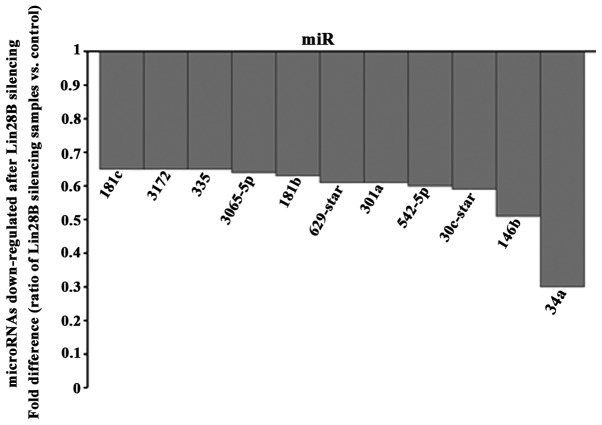
Fold difference (ratio of Lin28B silencing samples vs. control) of microRNAs downregulated in the DU145 AIPC cells. MicroRNA profiling was determined by GeneChip-miRNA 2.0 array. A threshold of 1.5-fold change was used.

A microRNA profile (GeneChip hybridization) at 48 h upon transfection of DU145 prostate cancer cells using siRNA against Lin28B was determined. A threshold of 1.5-fold change up or down (ratio of silencing samples vs. control) was used for the final analysis. Among the 19 microRNAs found upregulated (Fig. 4) and 11 microRNAs downregulated (Fig. 5), miR-212 and miR2278 were the most upregulated. TargetScan (http://www.targetscan.org/) was used to detect which of these microRNAs found altered in this study can potentially target the messenger RNA of Lin28B and it revealed that among all of these microRNAs found altered; only miR212, miR-181b and miR-181c could target the messenger RNA of Lin28B. Because miR-212 was the most altered microRNA, it has never been linked functionally to Lin28B in any cancer, and miR-212 was found suppressed in prostate cancer tissues (13), we validated the upregulation of miR-212 by real-time PCR at 24, 48 and 72 h after the Lin28B silencing and the maximum upregulation of miR-212 was detected at 24 h (fold difference, 440.93). A test for contrast used revealed that the upregulation of miR-212 was significant at all the time points analyzed (Table I).

### Lin28A and Lin28B have structural differences

**Figure 6:**
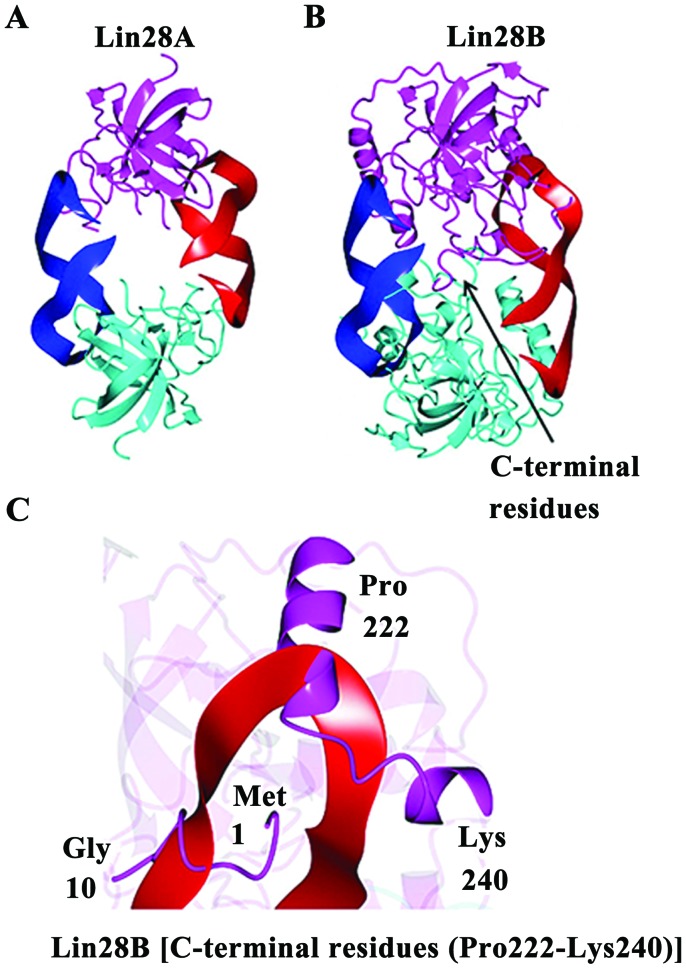
(A) Crystal structure of Lin28A:let7d complex (PDB:3TRZ). The complex consists of 2-fold symmetric Lin28A subunits (magenta/cyan) that coordinate the miRNA (red/blue) via interaction with the Lin28A CSD and ZNF domains. (B) Lin28B structure predicted from I-TASSER colored as in panel A. C-terminal residues of the putative Lin28B dimer are indicated by the arrow. (C) Zoomed-in view of the C-terminal residues (Pro 222-Lys 240) downstream of the ZNF2 domain as well as the N-terminal residues (Met1 to Gly 10) that would need to adopt a different conformation.

Lin28B shows unique microRNA binding characteristics. The Lin28B structure predicted from I-TAS-SER is shown in Fig. 6. A 2-fold symmetric model was generated by the superposition of the Lin28A structure (Fig. 6A) onto the I-TASSER model of Lin28B. If Lin28B were to bind miRNAs in a manner similar to Lin28A, conformational changes would be necessary, relative to the I-TASSER model, to prevent steric clashes between C-terminal residues of the putative Lin28B dimer (Fig. 6B). The C-terminal residues (Pro 222-Lys 240) downstream of the ZNF2 domain as well as the N-terminal residues (Met1 to Gly 10) would also need to adopt a different conformation than that observed for the I-TASSER model of Lin28B in order to accommodate RNA binding or the RNA would need to adopt a different binding mode relative to the Lin28A structure (Fig. 6C).

## Discussion

**Figure 7:**
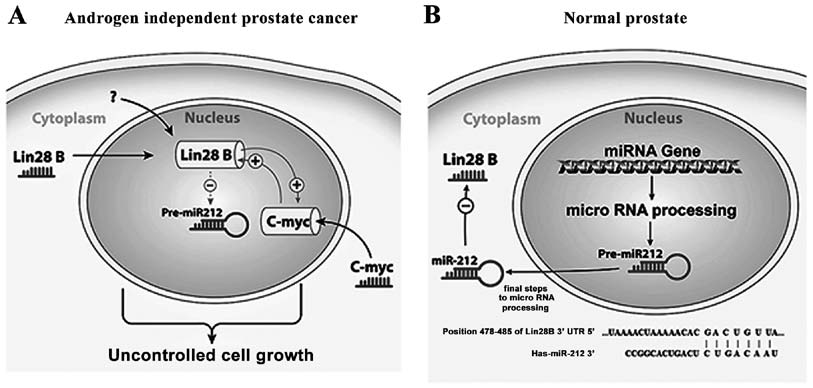
Model of the potential regulatory loop between Lin28B and miR-212. (A) In androgen-independent prostate cancer. (B) Normal prostate.

We report novel findings regarding the expression, function and structure of Lin28B and its connection to the microRNA pathways in AIPC. Lin28B is a member of the Lin28 protein family, whose gene is localized on chromosome 6. Lin28B expression has recently been detected in the nuclei and cytosol of prostate cancer cells; with no substantial changes in its expression with increasing tumor aggressiveness as measured by Gleason score (5). Similarly, we found Lin28B is expressed in prostate adenocarcinomas (irrespective of Gleason grade) but not in the glands of normal prostate tissue. In addition, we found strong nuclear staining in the nuclei and in the cytosol of DU145 prostate cancer cells. Lin28B has been presented to be tumorigenic in a prostate cancer mouse model (10) but the role of Lin28B in androgen-independent prostate cancer is unidentified. MicroRNA profiling in the androgen-independent prostate cancer cell line DU145 performed after silencing Lin28B by siRNA revealed up- and downregulations of several microRNAs with these most alterated being miR-212 and miR-2278. MiR-212 upregulation correlated with a decreased Lin28B messenger RNA and protein levels upon Lin28B silencing. This inverse relationship between Lin28B and miR-212 and its implication in prostate cancer development has not been previously reported. Our findings correspond with a recent publication by Walter and colleagues demonstrating the absence of miR-212 in prostate tumors, as compared to its normal expression in neighboring normal epithelium and/or stroma (13). In addition, miR-212 down-regulation has been described in lung cancer and is associated with the severity of the disease (14). TargetScan predictions display that Lin28B messenger RNA may be a target of miR-212 but not a target of miR-2278. Based on our results, we hypothesize that Lin28B inhibits the tumor suppressor activity of miR-212 in AIPC (Fig. 7A), potentially playing a role in prostate carcinogenesis or progression, while in normal prostate miR-212 suppresses the expression of Lin28B by targeting it mRNA (Fig. 7B). The published data obviously suggest an important role of miR-212 in the carcinogenesis process in general, but the microRNA profile we analyzed shows Lin28B as an oncogene regulator involving more than one pathway. Some of the microRNAs upregulated upon Lin28B silencing have never been associated with Lin28B. TargetScan did not predict them as biological Lin28B targets (by searching for the presence of conserved sites). It is possible that Lin28B knock/down activates other molecular pathways not yet described, inducing alteration of these microRNAs in prostate cancer cells, relating them to Lin28B.

Correlating well with previous data, miRNAs found to be upregulated upon Lin28B silencing in our present study include miR-212, downregulated in prostate cancer (13), and miR-146 which has been reported to suppresses tumor growth and progression in castration-resistant prostate cancer (15). Additionally, miR-1246 and miR-1308, are potential diagnostic biomarkers in multiple myeloma (16), and miR-1290 has been linked with the estrogen receptor-positive breast cancer phenotype (17).

Differing to our data, miR-4281, miR29b-1, miR-2861 have been reported to be upregulated in malignant melanoma (18), head and neck cancers (19) and thyroid cancer (20). Conversely, in our study they were only upregulated upon Lin28B silencing. This was also true for miR-1915 and miR-663, which have been associated with drug-therapy resistance in colorectal carcinoma (21) and breast cancer (22). MiR-4298 has been linked with the uncontrolled growth of gastric cancer cells (23). MiR-886-5p is an inhibitor of apoptosis in cervical cancer cells (24). Additionally, miR-149 was linked to the inhibition of non-small cell lung cancer (25) and miR-155 has been reported as a prognostic marker in chronic lymphocytic leukemia (25).

Lin28B has been linked to the negative targeting of the let-7 family of microRNAs in cancer and in prostate cancer (10,26) but, we did not find any evidence that the silencing of Lin28B in the DU145 AIPC cells affected any members of the Let7 microRNA family (data not shown).

In addition, eleven microRNAs were downregulated after Lin28B was silencing. MiR-335 (13) and miR-181c (27) have been found to be upregulated in prostate and gastric cancers, respectively. MiR-30e induces invasiveness of human glioma cells (28). Contradictorily, miR-34a overexpression was associated with the inhibition of prostate cancer cell growth (29), and miR-181b and miR-146b-5p have also been reported to be downregulated in prostate cancer (30,31). Also, miR-542-5p, reported as a tumor suppressor in neuroblastoma (32), was downregulated. A polymorphism at miR-629 was connected to the increased risk of lung cancer in Southern and Eastern Chinese population (33). Finally, from the thirty microRNAs found altered in this study, the biological functions of miR-2278, miR-1972, miR-1272, miR-3141 and miR-3172 have never been reported. These results need to be further validated to find each of the potential oncogenic pathways as it pertains specifically to prostate cancer development.

Human Lin28B is a 27-kDa protein that has been implicated in messenger RNA and microRNA binding (7,34). Lin28B shares highly sequence identity with Lin28A whose structure has been determined in complex with microRNAs (35). The resulting model using I-TASSER in our study suggests that Lin28B contains CSD/ZNF domains, similar to Lin28A, which one would expect to be implicated in the binding of nucleic acids as well. However, it also shows unique microRNA binding characteristics for Lin28B. If Lin28B were to bind miRNAs in a manner similar to Lin28A, conformational changes would be necessary to prevent steric clashes in the C-terminal and linker regions between the CSD and ZNF domains.

Our results provide additional data on the function of Lin28B and its interaction with microRNAs in AIPC. Our work also suggests structural differences between Lin28A and Lin28B, and a specific and unique interaction between Lin28B and microRNAs. We are proposing for the first time a novel oncogenic pathway in prostate carcinogenesis, involving Lin28B expression, miR-212 downregulation and activation of c-Myc oncogenic programs leading to the development of androgen-independent prostate cancer. There are few publications addressing the role of Lin28B in prostate cancer (10,36–38), and there are no publications addressing the function of Lin28B-microRNAs (different than let7)-c-Myc pathway in prostate cancer. To date, there are no reports in the literature regarding the function of miR-2278 in cancer or other disease states. But, our data using target scan shows no reciprocity between Lin28B:miR-2278. Therefore, for the first time; we are reporting a potential regulatory loop formed between Lin28B:miR-212 to regulate c-Myc in AIPC. This work may lead to the identification of a unique role of these molecules in the prostate cancer. Furthermore, the elucidation of the Lin28B-miR-212-c-Myc pathway may lead to new therapeutic approaches in the management of androgen-independent prostate cancer.

For the first time, we are proposing a unique nucleotide binding feature for Lin28B. Lin28B protein was found overexpressed in prostate adenocarcinoma tissue, regardless the grade or Gleason score, and in prostate cancer cell lines but not in normal prostate cancer tissues. We are showing an oncogenic pathway in prostate cancer of Lin28B overexpression involving miR-212 downregulation and increased levels of c-Myc protein, but not c-Myc messenger RNA. The messenger RNA of Lin28B was predicted to be a target of miR-212. Our findings open a new possible avenue for the study, understanding and treatment of androgen-independent prostate cancer. More studies are needed to further characterize this Lin28B-miR-212-c-Myc oncogenic pathway.

## Figures and Tables

**Table I:** Validation of the downregulation of miR-212 at 24, 48 and 72 h upon Lin28B silencing in the DU145 androgen-independent prostate cancer cell line using real-time PCR.

Sample	Δ ΔCT = ΔCT treated - ΔCT untreated	Fold difference: miR-212 expression relative to control 2^(−Δ ΔCT)^	Test for contrast
siLin28B			
24 h	−8.78	440.93	F=6178.7; df=1, 12; p<0.001
48 h	−3.78	13.69	F=15.65; df=1, 26; p<0.001
72 h	−3.73	13.269	F=93.32; df=1, 15; p<0.005
